# Genomic analysis of the inbreeding load for body weight, carcass and reproductive traits in the Rubia Gallega beef cattle population

**DOI:** 10.1186/s12711-026-01039-8

**Published:** 2026-03-13

**Authors:** Carlos Hervás-Rivero, David López-Carbonell, Manuel Sánchez-Díaz, Luis Varona

**Affiliations:** Facultad de Veterinaria, Instituto Agroalimentario de Aragón (IA2), Universidad de Zaragoza, 50013 Zaragoza, Spain

## Abstract

**Background:**

Inbreeding, resulting from mating between relatives, leads to inbreeding depression, which can be traced back to hidden ancestral inbreeding loads. These loads exhibit variability and act as additive genetic effects that are only expressed in the inbred offspring. The objective of this study was to quantify the variance of the inbreeding loads and its correlation with additive genetic effects for seven traits in the Rubia Gallega population: birth weight, weaning weight, cold carcass weight, carcass conformation, carcass fatness, calving interval, and age at first parity. A single-step GBLUP Bayesian analysis was used by a Gibbs sampler. Additionally, the equivalence between GBLUP and SNP-BLUP was used for locating the genomic regions associated with the highest variances.

**Results:**

The pedigree included 522,885 animals, of which 4984 were genotyped with the Axiom_BovMDv3 chip. A total of 246,393 individuals were inbred, with an average inbreeding coefficient of 0.044 ± 0.059, attributed to 4712 ancestors through 9.8 million partial inbreeding contributions. The estimated proportion of phenotypic variance explained by inbreeding loads for an inbreeding coefficient of 0.10 ranged from 0.012 (Birth weight) to 0.101 (Weaning weight), consistently below the heritabilities of the traits. Genetic correlations between inbreeding load and additive effects were always negative. The average prediction accuracy for inbreeding-load effects in young selection candidates was low and exceeded 0.7 only in older animals. The genomic distribution of additive and inbreeding load variances was uneven, with some regions overlapping and others being specific to inbreeding load.

**Conclusions:**

This study demonstrates that the inbreeding load variance is low compared to the additive genetic variance across a range of growth, carcass, and reproductive traits. The results were also consistent with a previous study that theoretically demonstrated a negative correlation between additive effects and inbreeding load. The potential to purge deleterious alleles appears limited, largely due to the low prediction accuracy observed in young individuals. Nevertheless, the higher accuracy of inbreeding-load estimates in ancestral animals could still be exploited to guide the unavoidable inbreeding in small populations through informed mating strategies, thereby minimizing undesirable inbreeding-depression effects. The heterogeneous genomic distribution of the inbreeding load suggests new opportunities for identifying genes in which deleterious or semideleterious alleles may be located.

**Supplementary Information:**

The online version contains supplementary material available at 10.1186/s12711-026-01039-8.

## Background

Inbreeding occurs when related individuals mate and is often associated with inbreeding depression, defined as a decline in phenotypic performance [1]. This phenomenon has been widely documented across animals, plants, and humans [1, 2]. Inbreeding depression results from increased homozygosity in inbred individuals, which can lead to the loss of heterozygote advantage (overdominance) or an increased expression of deleterious recessive alleles. Recessive alleles that remain masked in the heterozygous state contribute to an individual’s inbreeding load, which, when revealed through inbreeding, gives rise to inbreeding depression. This inbreeding load varies among founders [3, 4] and has been shown to be a heritable additive trait, proportional to the number of recessive alleles carried by an individual [5].

Inbreeding loads are only expressed in inbred individuals. However, in complex pedigrees, each inbred individual may inherit inbreeding contributions from multiple ancestors. These ancestral contributions can be decomposed through Mendelian partitioning [6], enabling their incorporation into a linear mixed model [7, 8] to predict individual inbreeding loads and to estimate their variance as well as their genetic correlation with additive genetic effects. This methodology has been successfully applied using phenotypic and pedigree information in beef cattle [8], dairy cattle [9], pigs [10], horses [11] and dairy sheep [5]. The additive nature of inbreeding loads [5] also allows for their prediction using SNP data through a single-step GBLUP approach [12]. Furthermore, the equivalence between GBLUP and SNP-BLUP enables the estimation of SNP effects [13], facilitating the identification of genomic regions that contribute most to the variance of the inbreeding loads.

Therefore, the objective of this study is to gain a deeper understanding of the variability in inbreeding loads and their correlation with additive genetic effects for seven traits related to body weight (BW: birth weight; WW: weaning weight), carcass characteristics (CCW: cold carcass weight; CONF: carcass conformation; FAT: carcass fatness), and reproduction (CI: calving interval; AFP: age at first parity) in the Rubia Gallega beef cattle population. Additionally, the study aims to identify genomic regions that account for the greatest proportion of variance in inbreeding load.

## Materials and methods

### Data

The datasets used in the study included phenotypic and pedigree information collected by ACRUGA (Asociación Nacional de Criadores de Ganado Vacuno Selecto de Raza Rubia Gallega). Prior to analysis, outliers were removed by excluding observations that deviated more than ± 3 standard deviations from the mean. After quality control, the final phenotypic dataset comprised 369,226 records for BW, 113,869 for WW, 107,110 for CCW, 107,046 for CONF, 106,962 for FAT, 278,330 for CI, and 51,229 for AFP (see Table 1). Records for BW, CI, and AFP were available from 1980 to 2023; WW records from 1990 to 2023; and CCW, CONF, and FAT records from 2010 to 2023. The distribution of phenotypic records by birth year is shown in Additional file 1 Fig. S1. The average age for recording WW was 212.1 days, with a standard deviation of 19.3 days. For CCW, CONF and FAT, the average age for slaughter and recording was 275.5 days, with a standard deviation of 25.7 days. CONF was assessed using the SEUROP classification system [14] and converted into a numeric scale from 1 (P- poorly conformed carcass) to 18 (S+ excellently conformed carcass). Carcass fatness (FAT) was visually scored by trained technicians on a scale from 1 (low fat content) to 15 (high fat content).

**Table 1:** Number of records (N), phenotypic mean (Mean), and standard deviation (SD) for body weight, carcass and reproductive traits in the Rubia Gallega population

Trait	*N*	Mean	SD
BW	369,226	42.65 Kg	7.02 Kg
WW	113,869	287.87 Kg	47.71 Kg
CCW	107,110	224.58 Kg	41.23 Kg
CONF	107,046	10.95 Units	1.99 Units
FAT	106,962	5.76 Units	1.33 Units
CI	278,330	401.42 Days	61.74 Days
AFP	51,229	865.85 Days	136.38 Days

*BW* Birth weight, *WW* Weaning weight, *CCW* Cold carcass weight, *CONF* Carcass conformation (CONF), *FAT* Carcass fatness, *AFP* Age at first parity, *CI* Calving interval.

The pedigree included 522,885 individual-sire-dam entries. The sire and dam were identified for 72.94% and 88.09% of the individuals, respectively. The pedigree depth and individual inbreeding was calculated with the “pedigree” R package [15]. The average depth was 8.61 generations with a standard deviation of 4.75 generations. There were 246,393 inbred individuals, with an average inbreeding of 0.044 and a standard deviation of 0.059. The distribution of inbreeding coefficients is presented in Table 2.

**Table 2:** Distribution of inbreeding coefficients in the Rubia Gallega population

Inbreeding coefficient	Number of individuals
0	276,492
0-0.05	185,152
0.05–0.10	35,115
0.10–0.15	11,558
0.15–0.20	3672
0.20–0.25	495
0.25–0.30	9268
0.30–0.35	805
0.35–0.40	253
> 0.40	75

A total of 4984 individuals were genotyped with the Axiom Bovine platform (Axiom_BovMDv3) from ThermoFisher Scientific (Waltham, MA, USA). Standard SNP quality control was performed using the preGSf90 software [16], excluding markers with a genotype call rate below 95% and those with a minor allele frequency (MAF) less than 0.05. Only SNPs located on autosomal chromosomes were retained, yielding a final dataset of 39,569 markers of 4960 individuals. Among them, 4351 individuals were inbred, with an average inbreeding coefficient of 0.035 ± 0.038, and 226 showed an inbreeding coefficient above 0.10, reaching a maximum of 0.383. The genotyped population included 889 sires, 3,620 dams, and 451 non-reproductive animals. The birth years of genotyped individuals (Additional file 1 Fig. S2) ranged from 1972 to 2023. Individuals born between 1972 and 2000 were sparsely represented and consisted mostly of historical AI sires. For animals born between 2000 and 2015, genotyping targeted individuals that were expected to have a major genetic impact on the population, primarily sires and dams of sires. Since 2016, genotyping capacity has increased substantially, enabling the routine genotyping of a larger number of dams, nearly all sires, and some non-reproductive individuals. The distribution of genotyped animals by reproductive category and the availability of phenotypic records are summarised in Table 3.

**Table 3:** Number of genotyped individuals by reproductive category (sires, dams, and non-reproductive animals) and phenotypic data availability for body weight, carcass and reproductive traits in the Rubia Gallega population

	BW	WW	CCW	CONF	FAT	AFP	CI
Sires	857	498	–	–	–	–	–
Dams	3528	2444	–	–	–	3492	3306
Non-Reproductive	442	329	153	153	153	–	–
TOTAL	4827	3271	153	153	153	3492	3306

*BW* Birth weight, *WW* Weaning weight, *CCW* Cold carcass weight, *CONF* Carcass conformation (CONF), *FAT* Carcass fatness, *AFP* Age at first parity, *CI* Calving interval.

It is important to note that the number of genotyped individuals is limited for some traits (CCW, CONF, and FAT). Nevertheless, the distribution of genetic variance across the genome for both the additive genetic component and the inbreeding load can still be reliably estimated, given the large number of progeny records available for sires and dams, as shown in Additional file 2 Table S1.

## Calculation of partial inbreeding coefficients

The Mendelian decomposition of inbreeding [6] resulted in a total of 9,795,019 partial inbreeding coefficients. On average, each inbred individual had 39.94 partial coefficients, with a standard deviation of 31.45. A large proportion (85.39%) of these coefficients were very low (< 0.001), and only 168,369 were over 0.01, accounting for just 1.73% of all coefficients. All partial inbreeding coefficients are generated by only 4,712 ancestors, each of whom contributed to inbreeding of an average of 2,078.74 individuals, with a standard deviation of 14,479.25. Among the ancestors, 700 were genotyped with the Axiom_BovMDv3 chip.

The distribution of the number of inbred individuals per ancestor was highly asymmetrical: 42 ancestors contributed to the inbreeding of more than 100,000 individuals, and 134 ancestors affected more than 10,000. In contrast, 1,811 ancestors contributed to the inbreeding of five or fewer individuals. The average number of individuals to which each ancestor contributes to inbreeding was strongly correlated with the age (year of birth) of the animals (Table 3). Furthermore, the proportion of genotyped animals was higher in more recent cohorts, as likewise shown in Table 4.

**Table 4:** Ancestral contributions to inbreeding by year of birth in the Rubia Gallega population

Year of Birth	*N*	%G	AN	SD
< 1981	936	0.64%	8622.50	29729.88
1981–1985	343	2.62%	2956.64	15385.02
1986–1990	482	2.69%	932.01	7260.52
1991–1995	685	5.10%	181.39	1178.08
1996–2000	645	8.53%	151.13	1379.83
2001–2005	650	27.53%	43.80	334.69
2006–2010	561	38.86%	15.40	59.83
2011–2015	331	41.09%	5.96	7.19
> 2015	79	62.03%	2.33	1.82

*N* Number of ancestors, %*G* Percentage of genotyped ancestors, *AN* Average number of individuals to which each ancestor contributes to inbreeding, *SD* Standard deviation the number individuals to which each ancestor contributes to inbreeding.

## Statistical models

The model used for the analysis of BW and AFP (Eq. 1) was:

1 $$\:\mathbf{y}=\mathbf{f}\mathrm{d}+\mathrm{Xb}+\mathrm{Wh}+\mathrm{Zu}+\mathrm{Ki}+\mathbf{e}$$

where **y** is the vector of phenotypic records, d is a regression coefficient total inbreeding, **b** is the vector of systematic effects, **h** is the vector of the random herd-year-season effects, respectively, **u** is the vector of the additive genetic effects, **i** is the vector of individual inbreeding loads and **e** is the vector of residuals. The vector **f** represents the genealogical inbreeding. Finally, **X**,** W**,** Z** and **K** are the corresponding incidence matrices. As proposed by Varona et al. [8], the **K** matrix were calculated as $\:\mathbf{K}=\mathbf{T}\left(\mathbf{I}-\mathbf{P}\right)$, where **T** contains the partial inbreeding coefficients derived from the Mendelian decomposition [6] and links the phenotypic data of inbred individuals to the inbreeding load of their ancestors that contributes to inbreeding. **P** is a matrix with zeros in the diagonal and 0.5 in the elements corresponding to the relationships between an individual and its sire and dam.

Some traits (WW, CCW, CONF, and FAT) were not recorded at a uniform age. Therefore, the model included an additional regression coefficient c, linked to the phenotypic data through the vector **t**, which contains the age at recording. The model (Eq. 2) was specified as follows:

2 $$\:\mathbf{y}=\mathbf{f}\mathrm{d}+\mathbf{t}\mathrm{c}+\mathrm{Xb}+\mathrm{Wh}+\mathrm{Zu}+\mathrm{Ki}+\mathbf{e}$$

Moreover, since CI was measured repeatedly for each dam, the model (Eq. 3) included an additional permanent environmental effect (***p****)*, associated with the phenotypic records through the incidence matrix ***L***:

3 $$\:\mathbf{y}=\mathbf{f}\mathrm{d}+\mathrm{Xb}+\mathrm{Wh}+\mathrm{Zu}+\mathrm{Ki}+\mathbf{L}\mathbf{p}+\mathbf{e}$$

The systematic effects included the overall mean, as well as sex (BW, WW, CCW, CONF, FAT), age of the dam (BW, WW), slaughterhouse (CCW, CONF, FAT) and parity order (CI). The number of levels for each systematic and random environmental effect is presented in Additional file 2 Table S2. The prior distributions of **h** and **p** were the following multivariate Gaussian distributions:

4 $$\:\mathbf{h}\sim\mathrm{N}\left(0,\mathbf{I}{\boldsymbol{\upsigma\:}}_{\mathbf{h}}^{2}\right)\,and\,\:\mathbf{p}\sim\mathrm{N}\left(0,\mathbf{I}{\boldsymbol{\upsigma\:}}_{\mathbf{p}}^{2}\right)$$

where $\:{\boldsymbol{\upsigma\:}}_{\mathbf{h}}^{2}$ and $\:{\boldsymbol{\upsigma\:}}_{\mathbf{p}}^{2}$ were the herd-year-season and permanent environmental variances, respectively. The prior distribution of the additive breeding values and inbreeding loads was defined as:

5 $$\:\left(\begin{array}{c}\mathbf{u}\\\:\mathbf{i}\end{array}\right)\mathrm{~}\mathrm{N}\left(\begin{array}{c}0\\\:0\end{array},\mathbf{V}\otimes\:\mathbf{H}\right)$$

with $$\:\mathbf{V}=\left(\begin{array}{cc}{{\upsigma\:}}_{\mathrm{u}}^{2}&\:{{\upsigma\:}}_{\mathrm{ui}}\\\:{{\upsigma\:}}_{\mathrm{ui}}&\:{{\upsigma\:}}_{\mathrm{i}}^{2}\end{array}\right)$$. $\:{\sigma\:}_{u}^{2}$ is the additive genetic variance, $\:{{\upsigma\:}}_{\mathrm{i}}^{2}$is the inbreeding load variance and $\:{{\upsigma\:}}_{\mathrm{ui}}$ is the covariance between them. Furthermore, **H** is the matrix that combines the genomic relationship matrix (**G**) [17] with the numerator relationship matrix (**A**) as described by Aguilar et al. [12]. The prior distribution of covariates (g, c), systematic effects (**b**) and variance components ($\:{{\upsigma\:}}_{\mathrm{h}}^{2}$, $\:{{\upsigma\:}}_{\mathrm{p}}^{2}$, **V**) were uniform within the parametric space. The analysis was performed through a Bayesian approach with a Gibbs sampler using a single long chain of 1,250,000 iterations, having discarded the first 250,000. The software used was developed for the study of Varona et al. [8].

## Accuracy estimation

The accuracy for each the *jth* additive genetic effect ($\:{acc(u}_{j})\:$and inbreeding load ($\:{acc(i}_{j})\:$is approximated as:

6 $$\:{\mathrm{a}\mathrm{c}\mathrm{c}\left(\mathrm{u}\right)}_{\mathrm{j}}=\sqrt{1-\frac{{\mathrm{P}\mathrm{S}\mathrm{D}(\mathrm{u}}_{\mathrm{j}})}{{\mathrm{H}}_{\mathrm{j}\mathrm{j}}{{\upsigma\:}}_{\mathrm{u}}^{2}}}\,and\,\:{\mathrm{a}\mathrm{c}\mathrm{c}\left(\mathrm{i}\right)}_{\mathrm{j}}=\sqrt{1-\frac{{\mathrm{P}\mathrm{S}\mathrm{D}(\mathrm{i}}_{\mathrm{j}})}{{\mathrm{H}}_{\mathrm{j}\mathrm{j}}{{\upsigma\:}}_{\mathrm{i}}^{2}}}$$

where $\:{\mathrm{P}\mathrm{S}\mathrm{D}(\mathrm{u}}_{\mathrm{j}})$ and $\:{\mathrm{P}\mathrm{S}\mathrm{D}(\mathrm{i}}_{\mathrm{j}})$ were the posterior standard deviation for the *jth* additive genetic effect and inbreeding load, $\:{\mathrm{H}}_{\mathrm{j}\mathrm{j}}$ is the diagonal element of the **H** matrix, and $\:{{\upsigma\:}}_{\mathrm{u}}^{2}$ and $\:{{\upsigma\:}}_{\mathrm{i}}^{2}$ were the posterior mean estimates of the additive genetic and the inbreeding load variance.

## Estimation of SNP effects

The posterior mean estimates of the breeding values (**u**) and the inbreeding loads (**i**) were used to compute the SNP effects (***s***_***u***_, ***s***_***i***_), following the approach proposed by Wang et al. [13]:

7 $$\:{\mathbf{s}}_{\mathrm{u}}=\frac{{\mathbf{W}}_{\mathbf{g}}^{\mathbf{{\prime\:}}}{\mathbf{G}}^{-1}\mathbf{u}}{\sum\:_{\mathrm{j}=1}^{\mathrm{N}}2{\widehat{\mathrm{p}}}_{\mathrm{j}}(1-{\widehat{\mathrm{p}}}_{\mathrm{j}})}$$

8 $$\:{\mathbf{s}}_{\mathrm{i}}=\frac{{\mathbf{W}}_{\mathbf{g}}^{\mathbf{{\prime\:}}}{\mathbf{G}}^{-1}\mathbf{i}}{\sum\:_{\mathrm{j}=1}^{\mathrm{N}}2{\widehat{\mathrm{p}}}_{\mathrm{j}}(1-{\widehat{\mathrm{p}}}_{\mathrm{j}})}$$

where **W**_**g**_ is the matrix containing gene content centred by subtracting twice the estimated allelic frequencies of each SNP of the population ($\:{\widehat{\mathrm{p}}}_{\mathrm{j}}$). The estimated SNP effects were then used to calculate the variances explained by each SNP as $\:{\widehat{{\upsigma\:}}}_{\mathrm{j}\mathrm{u}}^{2}=2{\widehat{\mathrm{p}}}_{\mathrm{j}}(1-{\widehat{\mathrm{p}}}_{\mathrm{j}}){\mathrm{s}}_{ju}^{2}$ and $\:{\widehat{{\upsigma\:}}}_{\mathrm{j}\mathrm{i}}^{2}=2{\widehat{\mathrm{p}}}_{\mathrm{j}}(1-{\widehat{\mathrm{p}}}_{\mathrm{j}}){\mathrm{s}}_{ji}^{2}$ for additive and inbreeding load effects, respectively. The additive ($\:{\widehat{{\upsigma\:}}}_{\mathrm{S}\mathrm{u}}^{2}$) and inbreeding load variances ($\:{\widehat{{\upsigma\:}}}_{\mathrm{S}\mathrm{i}}^{2}$) explained by a segment S (comprising a set of N SNPs) were computed as the sum of the corresponding SNP-wise variances:

9 $$\:{\widehat{{\upsigma\:}}}_{\mathrm{S}\mathrm{u}}^{2}=\sum\:_{\mathrm{j}=1}^{\mathrm{N}}{\widehat{{\upsigma\:}}}_{\mathrm{j}\mathrm{u}}^{2}$$

and.

10 $$\:{\widehat{{\upsigma\:}}}_{\mathrm{S}\mathrm{i}}^{2}=\sum\:_{\mathrm{j}=1}^{\mathrm{N}}{\widehat{{\upsigma\:}}}_{\mathrm{j}\mathrm{i}}^{2}$$

These calculations were carried out using the POSTGSF90 software, part of the BLUPF90 family of programs [16]. We estimated the proportion of additive genetic variance explained by genomic regions consisting of 25 and 50 consecutive SNP markers by the options ‘windows variance 25’ and ‘windows variance 50’, respectively. Genomic windows were defined by a fixed number of SNPs rather than a physical genomic distance to avoid potential spurious associations arising by heterogeneous SNP marker density across the genome [18]. Finally, genomic regions explaining more than 1% of additive or inbreeding load genetic variance were identified, and the genes located within those windows were explored using the Biomart tool (www.ensembl.org) [19], which hosts the latest bovine genome assembly, *Bos taurus* (ARS-UCD2.0).

## Results

### Variance component estimation

Table 5 presents the posterior mean estimates and the posterior standard deviations for the average inbreeding depression and variance components for all traits.

**Table 5:** Posterior estimates of inbreeding depression and variance components for for body weight, carcass and reproductive traits in the Rubia Gallega population

	BW	WW	CCW	CONF	FAT	CI	AFP
d	− 1.20 (0.33)	− 33.14 (4.97)	− 59.29 (3.91)	− 0.88 (0.22)	− 0.86 (0.14)	30.24 (6.38)	74.27 (34.11)
D(%-M)	2.81% (0.77%)	11.51% (1.72%)	26.40% (1.74%)	8.01% (2.01%)	14.93% (2.43%)	7.53% (1.59%)	8.58% (3.93%)
(D%-SD)	17.09% (4.70%)	69.46% (10.35%)	143.80% (9.48%)	44.22% (11.06%)	64.66% (10.52%)	48.98% (10.33%)	54.45% (25.01%)
$\:{{\upsigma\:}}_{\mathrm{u}}^{2}$	9.27 (0.16)	450.64 (14.28)	476.50 (13.04)	1.43 (0.04)	0.58 (0.02)	199.36 (12.25)	4,298.96 (240.37)
$\:{{\upsigma\:}}_{\mathrm{i}}^{2}$	57.97 (18.35)	1,907.93 (3,513.18)	7,965.10 (2,401.53)	25.61 (7.18)	10.78 (3.13)	7,356.46 (3,544.73)	194,546.51 (80,158.54)
$\:{{\upsigma\:}}_{\mathrm{ui}}$	− 11.03 (2.57)	− 547.68 (268.18)	− 847.18 (259.82)	− 3.26 (0.84)	− 1.85 (0.37)	− 460.15 (294.60)	− 12,964.96 (6,833.75)
$\:{{\upsigma\:}}_{\mathrm{h}}^{2}$	7.89 (0.23)	251.86 (9.80)	302.00 (11.88)	0.27 (0.01)	0.10 (0.01)	264.29 (9.79)	2,414.99 (113.29)
$\:{\sigma\:}_{p}^{2}$	–	–	–	–	–	208.33 (10.62)	–
$\:{\sigma\:}_{e}^{2}$	29.49 (0.12)	980.22 (9.88)	456.65 (8.91)	1.59 (0.03)	1.01 (0.01)	3,080.66 (9.49)	11,985.86 (186.16)

*BW* Birth weight, *WW* Weaning weight *CCW* Cold carcass weight, *CONF* Carcass conformation, *FAT* Carcass fatness, *AFP* Age at first parity, *CI* Calving interval, *d* Average inbreeding depression in phenotypic units for fully inbred individuals (F = 1), *D*%-*M* Average inbreeding depression in percentage of the phenotypic mean for fully inbred individuals, *D*%-*SD* Average inbreeding depression in percentage of the phenotypic standard deviation for fully inbred individuals, $\:{\sigma\:}_{u}^{2}:\:$ Additive genetic variance, $\:{\sigma\:}_{i}^{2}$ Inbreeding load variance, $\:\:{\sigma\:}_{\mathrm{ui}}$ Covariance between the additive genetic and the inbreeding load effects, $\:{\sigma\:}_{h}^{2}$ Variance of the herd-year-season effects, $\:{\sigma\:}_{p}^{2}$ Permanent environmental variance, $\:{\sigma\:}_{e}^{2}$ Residual variance.

The average inbreeding depression (*d*) was negative for BW, W210, CCW, CONF, and FAT, and positive for CI and AFP. The 95% highest posterior density (HPD95%) intervals for all traits did not include zero. These estimates of average inbreeding depression show that inbreeding is consistently associated with reduced phenotypic performance across all traits, ranging from a 2.8% decrease in the phenotypic mean for BW to a 26.4% decrease for CCW and between a 17.09% (BW) to a 143.80% (CCW) with respect to the phenotypic standard deviation. Notably, the estimates of inbreeding depression for AFP and CI are positive, indicating that inbred individuals tend to exhibit longer AFP and CI.

**Fig. 1:**
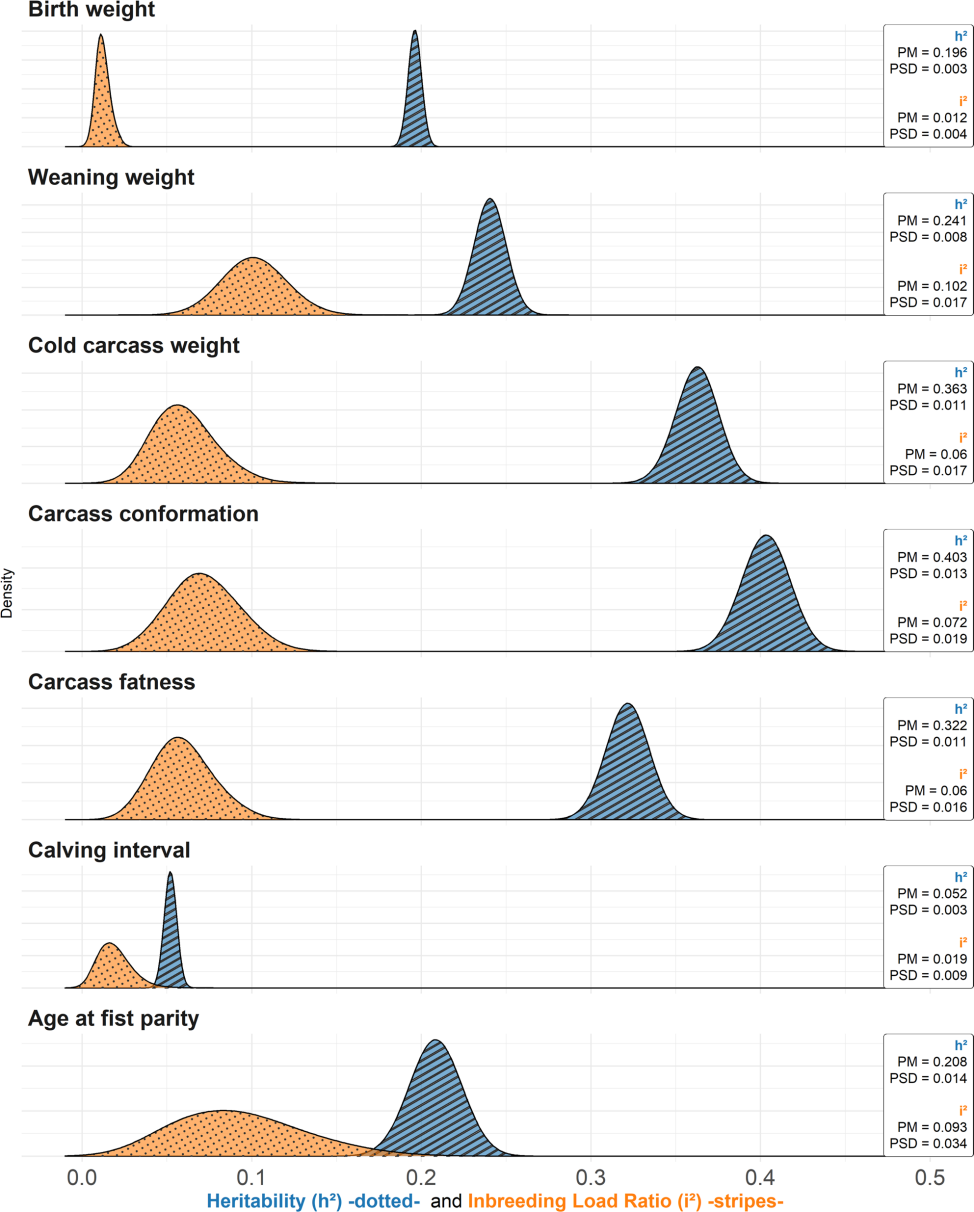
Posterior distributions of the ratios of inbreeding load variance to phenotypic variance (assuming an inbreeding coefficient of 0.10), and of heritabilities for birth weight, weaning weight, cold carcass weight, carcass conformation, carcass fatness, calving interval, and age at first parity in the Rubia Gallega population

The posterior mean estimates of the inbreeding load variances were consistently higher than those of the additive genetic variances (e.g., 57.97 vs. 9.27 for BW). However, it is important to note that these estimates correspond to the variance in inbreeding depression expected for a fully inbred descendant from a single ancestor [8], and thus are not directly interpretable. Accordingly, the estimates need to be rescaled to a defined inbreeding coefficient. To illustrate their magnitude, we calculated the ratio of the inbreeding-load variance to the phenotypic variance under a scenario of substantial inbreeding. For this purpose, we assumed an inbreeding coefficient of 0.10, which corresponds to the level observed in more than 25,000 individuals in the Rubia Gallega population. The posterior distributions of these ratios, together with the posterior distributions of trait heritabilities, are presented in Fig. 1.

As shown in Fig. 1, the posterior mean estimates of heritability ranged from 0.052 for CI to 0.403 for CONF, while the magnitude of the ratio of the inbreeding load variance, for an inbreeding coefficient of F = 0.10, was consistently lower than the trait heritability estimates, with posterior means ranging from 0.012 for BW to 0.101 for WW.

**Fig. 2:**
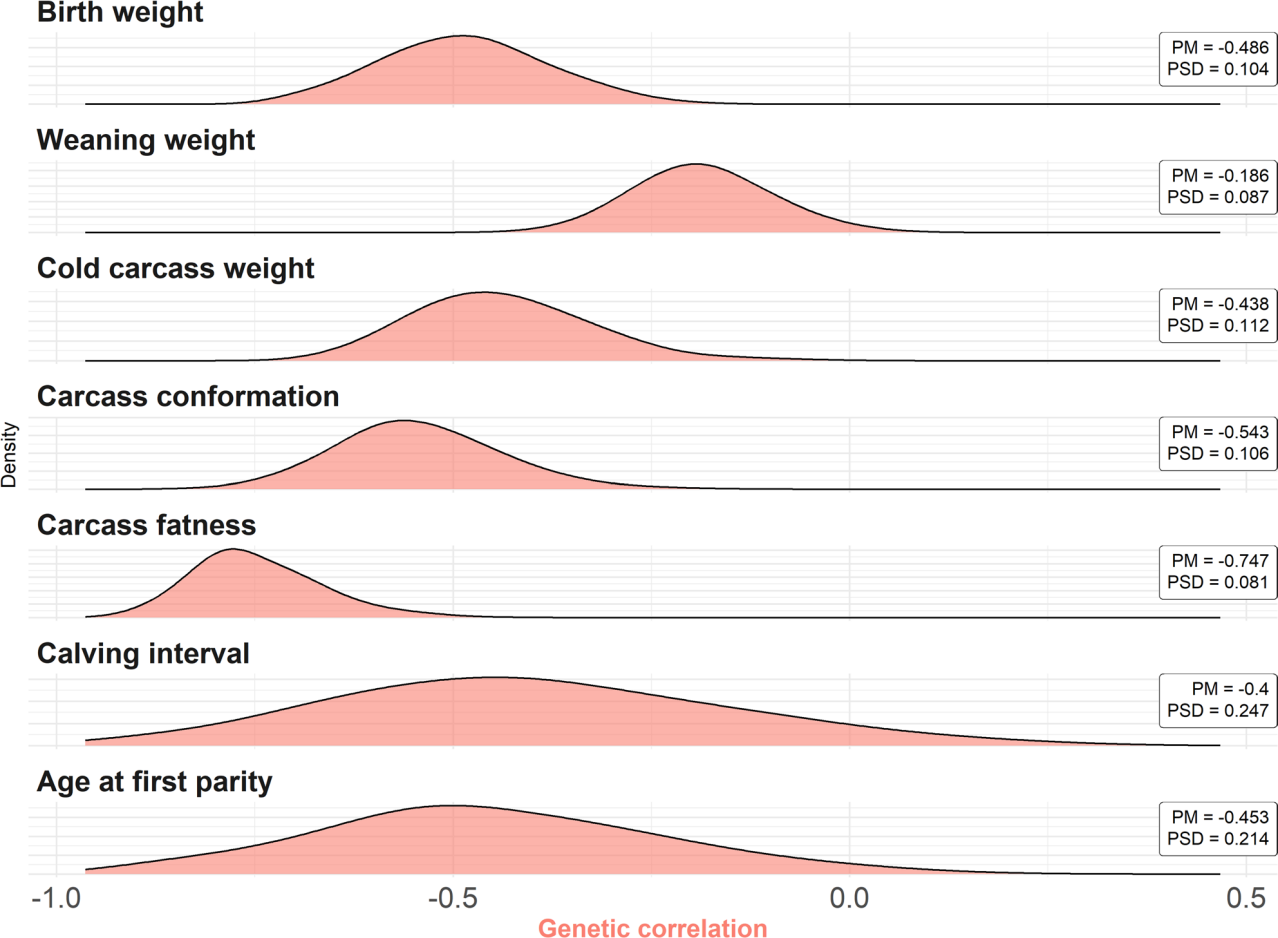
Posterior distributions of the genetic correlations between the inbreeding load and additive genetic effects for birth weight, weaning weight, cold carcass weight, carcass conformation, carcass fatness, calving interval, and age at first parity in the Rubia Gallega population

The posterior distributions of the genetic correlations between additive genetic effects and inbreeding load effects are shown in Fig. 2. Posterior mean estimates ranged from − 0.186 for WW to − 0.747 for FAT. The posterior probabilities of the genetic correlation being negative were 1.00 for BW, CCW, CONF, and FAT, and 0.983, 0.947, and 0.982 for WW, CI, and AFP, respectively.

**Fig. 3:**
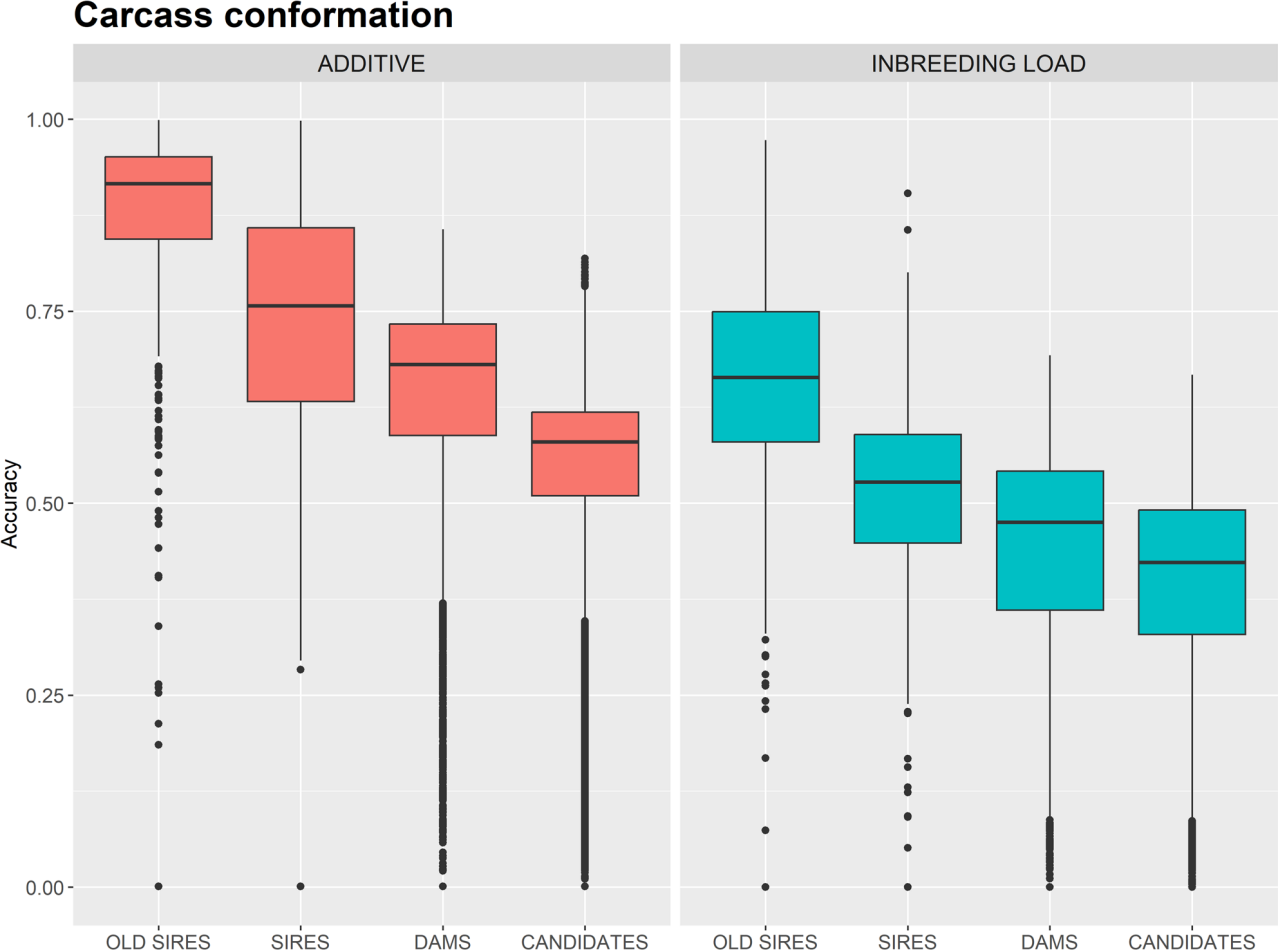
Boxplot of the estimated accuracy for carcass conformation for the additive genetic and the inbreeeding loads for old AI sires, current sires and dams and candidates to selection

*Accuracy of prediction of inbreeding loads* As is shown in Table 3, phenotypic data used to estimate inbreeding load are mostly available for older animals. As a result, predictions for more recent individuals rely predominantly on pedigree or genomic information, leading to reduced accuracies, even when genomic data are available [20]. This pattern is illustrated in Fig. 3, which presents a boxplot of prediction accuracies for the additive genetic effects and the individual inbreeding loads for carcass conformation (CONF) by four groups of individuals, old AI sires, available sires and dams and selection candidates. The results for the remaining traits are presented in Additional file 1 Fig. S3.

**Fig. 4:**
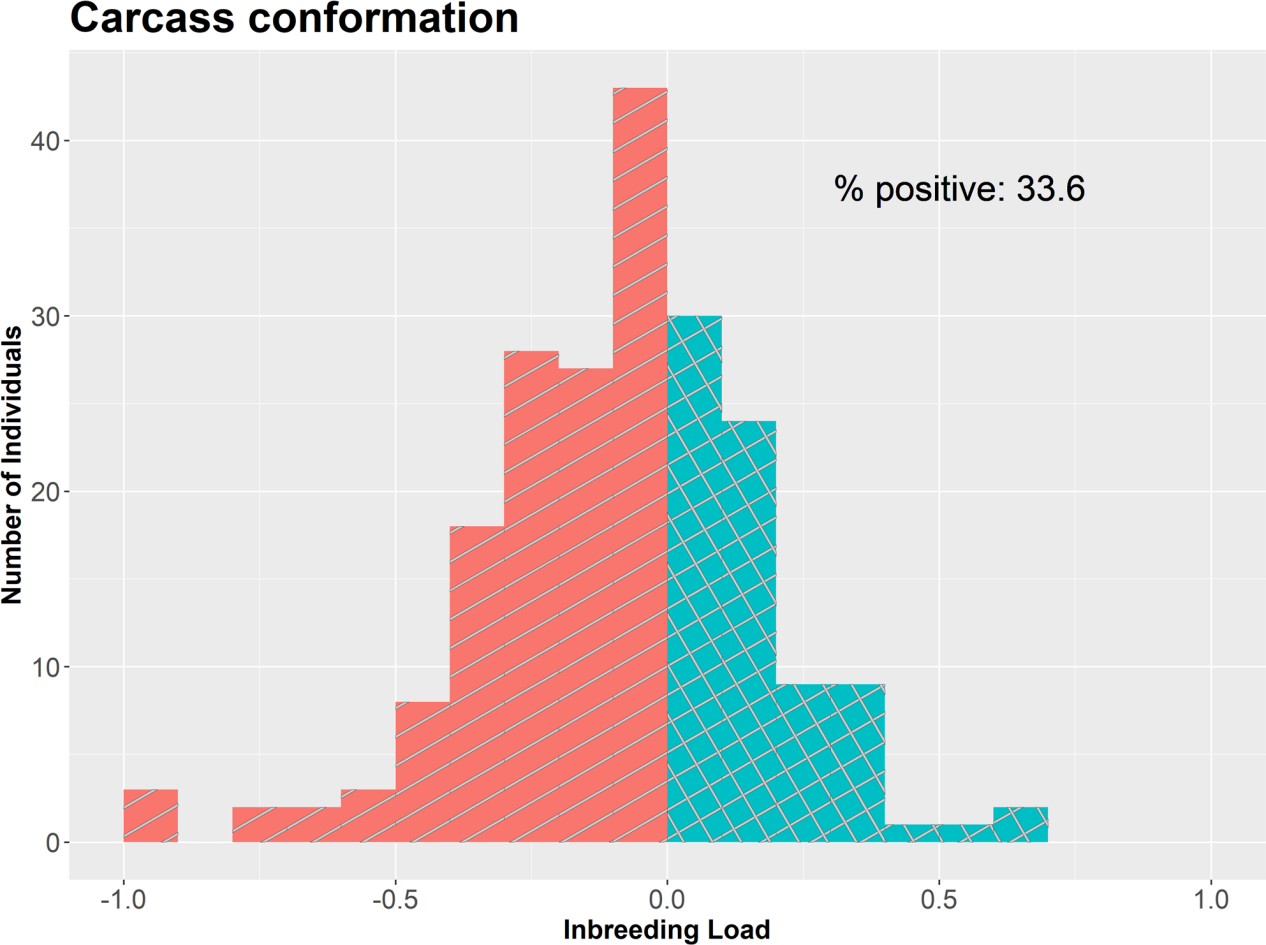
Histogram of the predictions of the inbreeding load for carcass conformation for individuals with accuracy over 0.7

The average prediction accuracies were lower for the inbreeding load than for the additive genetics effects for all groups of individuals and traits. For CONF, the average (± standard deviation) accuracy for the inbreeding load in candidates of selection was 0.39 (± 0.14), and in current sires and dams were 0.51 (± 0.13) and 0.44 (± 0.14), respectively. In contrast, the average accuracy for the inbreeding load of old AI sires was 0.65 (± 0.17), and a substantial percentage of them have accuracies over 0.7. The distribution of the predictions of the inbreeding loads for these individuals for CONF is presented in Fig. 4 for conformation and in Additional File 1 Fig. S4 for the remaining traits.

The results showed that 33.6% of individuals with an accuracy above 0.7 had a positive prediction of the inbreeding load for CONF. For the remaining traits, similar patterns were observed (Additional file 1 Fig. S4), ranging from 12.2% for CCW to 40.4% for BW.

**Fig. 5:**
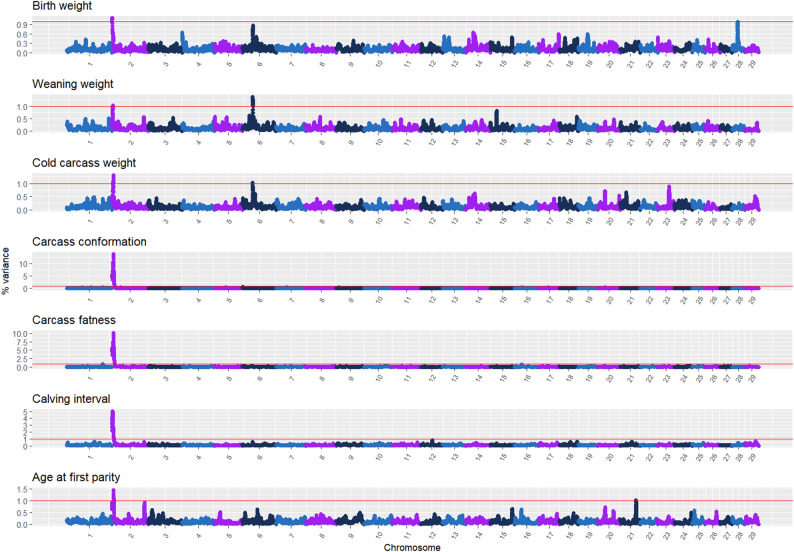
Manhattan plots of the percentage of the additive genetic variance explained by segments of 50 SNPs along the autosomal genome for birth weight, weaning weight, cold carcass weight, carcass conformation, carcass fatness, calving interval, and age at first parity in the Rubia Gallega population

*Distribution of the additive genetic variance* The Manhattan plots of the genomic scans representing the percentage of additive variance explained by segments of 25 and 50 SNP is presented in Fig. 5 and Additional file 1 Fig. S5, respectively.

**Fig. 6:**
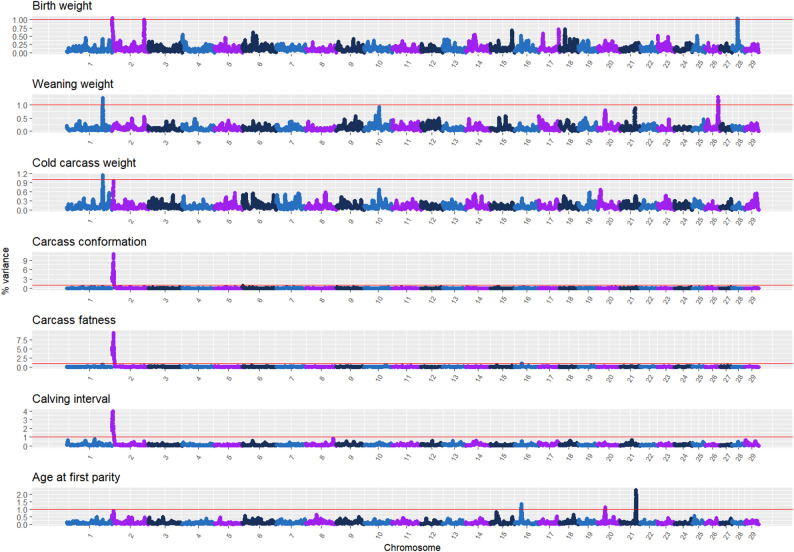
Manhattan plots of the percentage of the inbreeding load variance explained by segments of 50 SNPs along the autosomal genome for birth weight, weaning weight, cold carcass weight, carcass conformation, carcass fatness, calving interval, and age at first parity in the Rubia Gallega population

*Distribution of the inbreeding load variance* The Manhattan plots of the genomic scans representing the percentage of the inbreeding load variance explained by segments of 25 and 50 SNP is presented in Fig. 6 and Additional file 1 Fig. S6, respectively. 

## Discussion

The results obtained demonstrate that inbreeding has a clear and measurable effect on phenotypic performance in the Rubia Gallega breed. The negative inbreeding depression observed for BW, WW, CCW, CONF, and FAT, together with the positive effects detected for CI and AFP, indicates that growth, carcass, and fertility traits are adversely affected by increases in homozygosity. However, notable differences among traits arise when inbreeding depression is expressed as a percentage of the phenotypic mean or the phenotypic standard deviation, highlighting the heterogeneity in the estimated magnitude of inbreeding depression [2, 21]. In this study, the magnitude of the effects observed for growth traits—a 26.40% reduction of the mean and 143.80% of the phenotypic standard deviation for CCW, and an 11.51% reduction of the mean and 60.46% of the standard deviation for WW—was larger than the corresponding estimates for BW, and is consistent with previous findings in the literature [22]. Carcass conformation and fatness showed reductions of 8.01% and 14.93% of the phenotypic mean and 44.22% and 64.66% of the standard deviation, respectively, values that are in line with earlier estimates reported for these traits [23]. Reproductive traits are generally expected to be strongly affected by inbreeding depression [2]. Nevertheless, in this study AFP and CI showed only moderate increases in the phenotypic mean (7.53% and 8.58%, respectively), although they exhibited substantial percentages relative to the phenotypic standard deviation (48.98% and 54.45%, respectively).

The posterior mean heritability estimates were lowest for the reproductive trait CI, intermediate for BW, WW, and AFP, and highest for the carcass traits CCW, CONF, and FAT. These estimates fall within the ranges previously reported for this breed [24] and for other beef cattle populations [14, 25]. though the variance attributable to the inbreeding load exceeded the additive genetic variance, its relative contribution to the phenotypic variance—after scaling by the actual levels of inbreeding—was limited. The estimated ratio of inbreeding load variance for an inbreeding coefficient of F = 0.10 was consistent with earlier findings [5, 8, 9]. This result is expected because inbreeding depression is directly related to the magnitude of dominance variance [26], and aligns with dominance variance estimates reported in beef cattle [27, 28]. Overall, these findings indicate that incorporating inbreeding load into selection indices would likely have only a minor impact on selection decisions.

Strongly negative correlations were observed between additive and inbreeding load effects (e.g., up to − 0.747 for FAT). Following the development of Antonios et al. [5], the negative covariance between additive genetic effects and inbreeding load effects depends on the contribution of dominance gene action to the additive genetic variance. These estimates are also consistent with previous empirical findings [8]. However, the correlations observed in this study were more strongly negative than those reported for fertility traits in the Brown Swiss population [9] or for milk yield in sheep breeds [5]. This indicates that recessive alleles with favorable effects in heterozygous individuals contribute substantially to inbreeding depression. From a practical breeding perspective, this antagonistic relationship may complicate selection strategies, especially in closed or small populations where managing the accumulation of inbreeding is inherently difficult. Additive genetic improvement would entail a reduction in the inbreeding load, meaning that more phenotypic detrimental effects would be expressed in inbred individuals. Consequently, under selection, inbreeding depression is expected to become more pronounced in future generations.

Furthermore, as shown in Table 4, the number of descendants through which each ancestor contributes to inbreeding is much larger for older individuals. Consequently, the phenotypic information used to estimate individual inbreeding load is predominantly available for these older animals. The inbreeding load predictions for more recent individuals rely largely on pedigree or genomic information, resulting in reduced accuracy even when genomic data are available [20]. Consequently, the potential implementation of artificial purging is limited. Nevertheless, the accuracy of inbreeding load estimates for ancestral animals can play a strategic role, as it enables mating designs that channel inevitable inbreeding through individuals with the lowest inbreeding depression effects, or even through those exhibiting slight increases in phenotypic performance due to inbreeding. Thus, inbreeding load information provides a valuable tool for managing unavoidable inbreeding, allowing its redistribution toward less detrimental ancestors rather than simply avoiding or restricting inbreeding through methods such as minimum coancestry mating [29] or optimum contribution selection [30]. Moreover, predicting inbreeding loads allows these approaches to be combined with expectations of inbreeding depression, by restricting not the total inbreeding per se, but its phenotypic consequences.

Genomic analyses further revealed heterogeneous distributions of both additive and inbreeding load variances across the genome. The results for the additive genetic effect were consistent with those previously reported by [24], highlighting four genomic regions of particular interest. The most significant region is located on BTA2, surrounding the *MSTN* (*Myostatin*) gene, which accounts for more than 1% of the additive genetic variance across all seven analyzed traits. Its effect was especially pronounced for CONF (13.89%), FAT (10.20%), and CI (5.04%). It is well established that mutations in the *MSTN* gene segregate within the Rubia Gallega population [24, 31], and the strong phenotypic effects associated with these mutations [32, 33] are confirmed in the present study. A second prominent genomic region was identified on BTA6, between 37 and 40 Mb, explaining over 1% of the additive genetic variance for Weaning weight (WW) and Cold carcass weight (CCW). These results are consistent with the findings of Martínez-Castillero et al. [24]. This region has been widely reported to harbor QTLs associated with growth and carcass traits in beef cattle [33–35] and contains multiple candidate genes such as *LAP3* (*Leucine Aminopeptidase 3)*, *LCORL* (*Ligand Dependent Nuclear Receptor Corepressor Like*), and *NCAPG* (*Non-SMC Condensin I Complex Subunit G*). Two additional genomic regions (BTA1:132451691–132570640 and BTA21:57719367–58246444) were associated with more than 1% of the additive genetic variation in FAT and AFP, respectively. Both regions were identified by Martínez-Castillero et al. [24], and in their neighborhood there are several genes of interest that have been associated with economically relevant traits in beef cattle, such as the *NCK1* (*Cytoplasmic protein*) [36], the *RIN3* (*Ras and Rab Interactor 3)* [37] and the *LGMN* (*Legumain*) [38].

This results confirms that the inbreeding load is not uniformly distributed across the autosomal genome, as suggested by studies [39–42] exploring alternative approaches. Interestingly, several genomic regions were found to explain more than 1% of the inbreeding load variance despite having little to no impact on the additive genetic variance. These regions may harbor genes with recessive or non-additive effects that influence fitness-related traits. For instance, on chromosome BTA2 (127.4–127.6 Mb), a region associated with WW contains the *DUSP10* gene (*Dual Specificity Phosphatase 10*), which regulates the MAPK signaling pathway and plays a role in suppressing cell growth and proliferation. This gene has previously been linked to carcass weight and growth traits in cattle [43, 44]. Another relevant region was detected on BTA16 (25.2–25.3 Mb), associated with carcass fatness (FAT) and age at first parity (AFP). This region includes the *MIER3* (*Mesoderm Induction Early Response Protein 3*) gene, which has been reported to interact with *MSTN* and has emerged in GWAS as a candidate gene for survival traits in Sahiwal cattle [34] and fertility traits in Hanwoo cattle [45]. On BTA20 (22.1–22.3 Mb), in relation to AFP, we identified the *ADAM12* gene (*ADAM Metallopeptidase Domain 12*), known for its role in myogenesis and adipogenesis [46]. This gene has also been associated with body size in Belgian Blue cattle [47], suggesting a potential link to reproductive maturity and development. Weaning weight (WW) was also influenced by a region on BTA26 (45.4–47.3 Mb), which contains the *LRRTM3* (*Leucine Rich Repeat Transmembrane Neuronal 3*) gene. This gene has been implicated in skeletal and brain development, and although its role in growth is less direct, its function may relate to developmental robustness under inbreeding stress [48]. Finally, for birth weight (BW), a notable region on BTA28 (21.6–25.4 Mb) includes the *SIRT1* gene (*Sirtuin 1*), which has been linked to body weight, height, and other body measurements such as chest and hip width [49, 50]. Given its role in energy metabolism and growth regulation, SIRT1 may act as a modifier of early growth responses affected by inbreeding.

A previous study [42] investigating inbreeding depression was conducted in the same population but employing a completely different approach. That study evaluated performance differences between genotyped individuals with and without runs of homozygosity (ROH), whereas the present study associated inbreeding depression with the genotypes of the ancestors responsible for the inbreeding. Despite these methodological differences, both studies identified overlapping genomic regions on BTA1, BTA2, BTA6, and BTA21. The strongest concordance was observed on BTA2, encompassing the MSTN gene. In this region, detrimental effects on age at first calving (AFC) and early growth detected in the ROH analysis were corroborated in the present study across fertility, conformation, and weight traits. Interestingly, the genomic region on BTA6, which was associated with ROH in the previous study, was associated with additive genetic variance in the present analysis. This is not unexpected, as ROH may arise not only from inbreeding but also from selection acting on genomic regions harboring quantitative trait loci (QTL) [51]. Additionally, the genomic region on BTA21 was associated with ROH for WW in the previous study, whereas in the present analysis it was linked to inbreeding load for AFC and accounted for nearly 1% of the genetic variance in WW. Finally, the genomic region on BTA1 was associated with inbreeding load for WW and CCW in this study, while Mejuto-Vázquez et al. [42] identified it as an ROH-associated region for AFC.

In summary, the genomic scan identified regions of particular interest, some of which overlapped with those associated with additive genetic variance. Notably, the MSTN gene on BTA2 emerged as a major contributor to both inbreeding load and additive variance, reinforcing its known dominance effects related to double muscling and carcass traits [52]. Its impact was especially pronounced for CONF, FAT, and CI, reflecting its contribution to additive genetic variance for these traits. Additionally, regions previously reported on BTA1 and BTA21 accounted for more than 1% of the inbreeding load variance for WW and CCW, and for AFP, respectively. In contrast, the genomic region on BTA6 encompassing LCORL and NCAPG contributed substantially to additive variance but minimally to inbreeding load, indicating primarily additive effects. Other regions associated with inbreeding load but not with additive variance (e.g., DUSP10, MIER3, ADAM12, LRRTM3, SIRT1) suggest potential recessive deleterious effects that may affect robustness, development, fertility, or growth in inbred animals.

## Conclusions

This study demonstrates that inbreeding load variance is consistently lower than additive genetic variance across key growth, carcass, and reproductive traits in the Rubia Gallega population. The negative genetic correlations observed between inbreeding load and additive effects indicate that directional dominance contributes to the additive genetic variance. They further suggest that certain recessive alleles contributing to the observed inbreeding load do not impair phenotypic performance in the heterozygous state, but can exert deleterious effects when present in homozygosity, thereby leading to inbreeding depression. These findings underscore a fundamental trade-off that must be explicitly accounted for in the design of breeding programs. Although the low accuracy of inbreeding load estimates in young animals limits the potential for effective purging, accurate estimates in ancestral animals can be used to guide inevitable inbreeding in small populations, minimizing undesirable inbreeding depression effects. Furthermore, the heterogeneous genomic distribution of inbreeding load variance identifies specific regions potentially harboring deleterious alleles, providing valuable targets for future functional studies.

## Supplementary Information

Below is the link to the electronic supplementary material.

Additional File 1

Additional File 2

## Data Availability

Data will be available under reasonable request to the corresponding author.
