# Supplementary material for: Genomic analysis of the inbreeding load for body weight, carcass and reproductive traits in the Rubia Gallega beef cattle population
Source: Genet Sel Evol. 2026 Mar 13;58:21. doi: 10.1186/s12711-026-01039-8 (PMC13003673; doi:10.1186/s12711-026-01039-8)
Supplement: Supplementary file 1 — Additional File 1 [file 12711_2026_1039_MOESM1_ESM.pdf]

Genomic analysis of the inbreeding load for body weight, carcass and reproductive traits in the Rubia Gallega Beef Cattle population

Carlos Hervás-Rivero, David López-Carbonell, Manuel Sánchez-Díaz, Luis Varona*

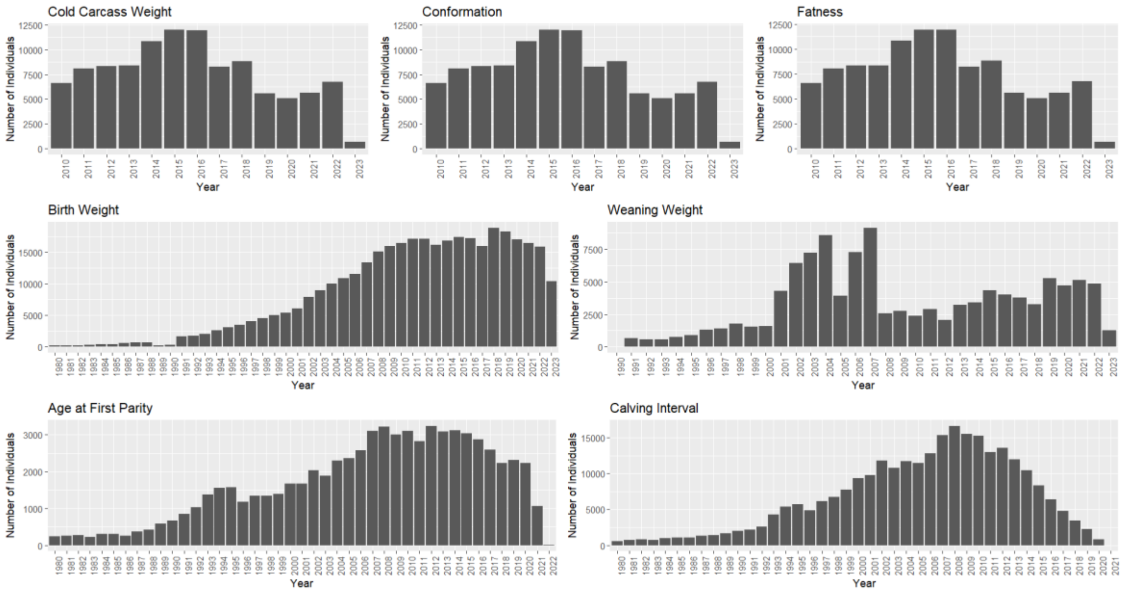

Supplementary Figure 1. Distribution of phenotypic records for Cold Carcass Weight, Conformation, Fatness, Birth Weight, Weaning Weight, Age at First Parity and Calving Interval across year of birth.

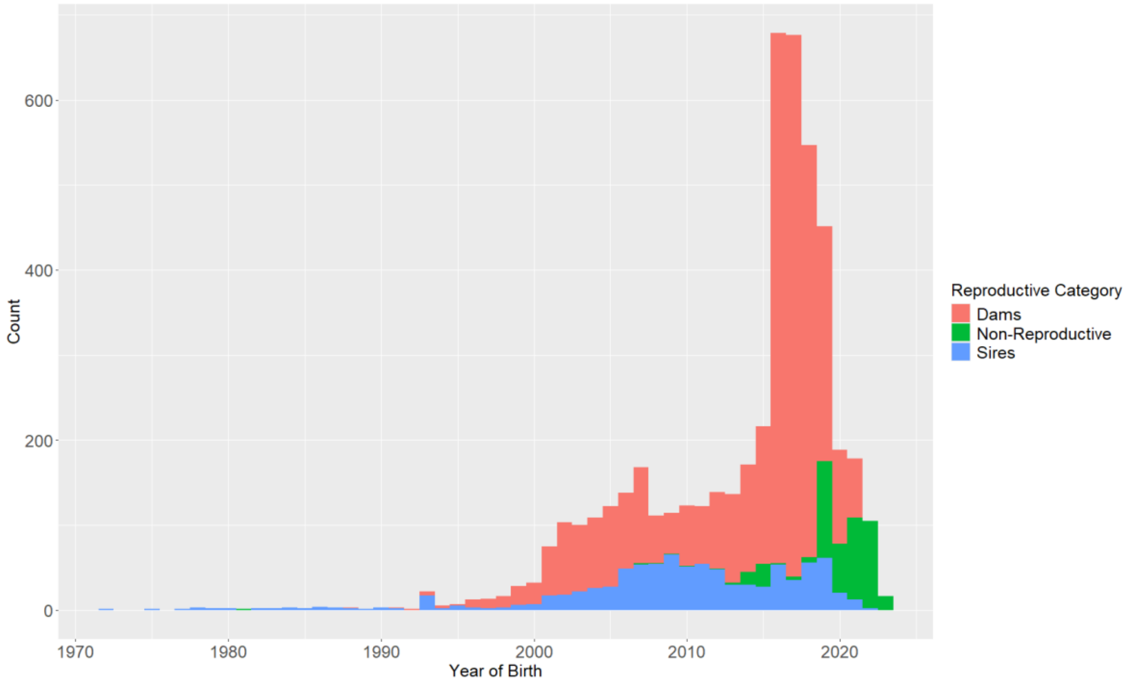

Supplementary Figure 2. Distribution of genotyped individuals across year of birth and by reproductive category (Sires, Dams and Non-Reproductive).

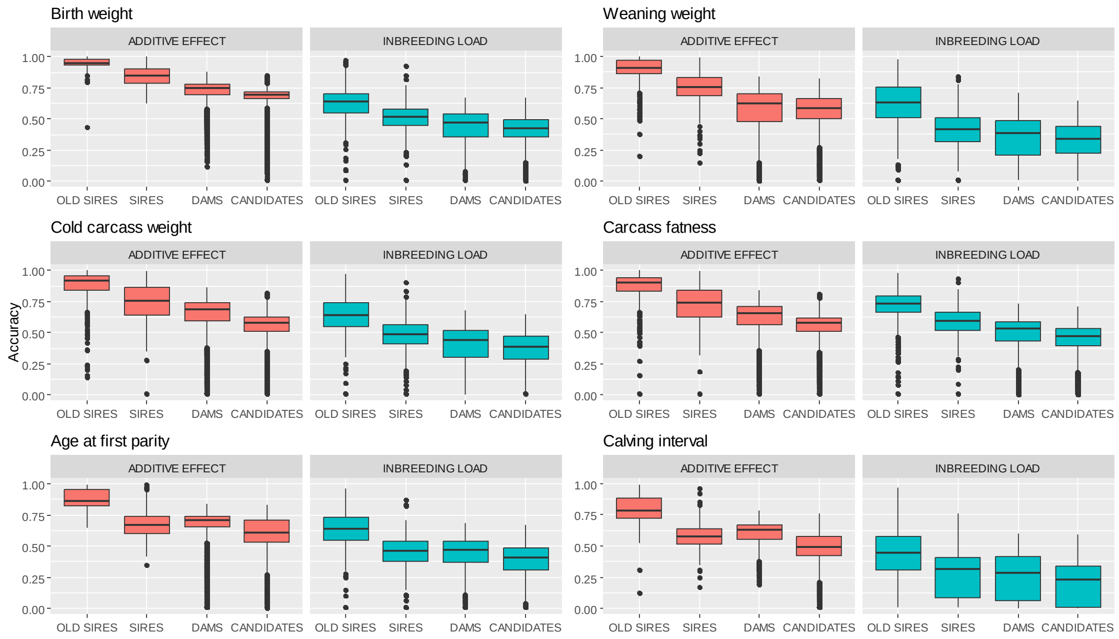

Supplementary Figure 3. Boxplot of the estimated accuracy for Birth Weight, Weaning Weight, Cold Carcass Weight, Fatness, Age at First Parity and Calving Interval for the additive genetic and the inbreeeding loads for old AI sires, current sires and dams and candidates to selection.

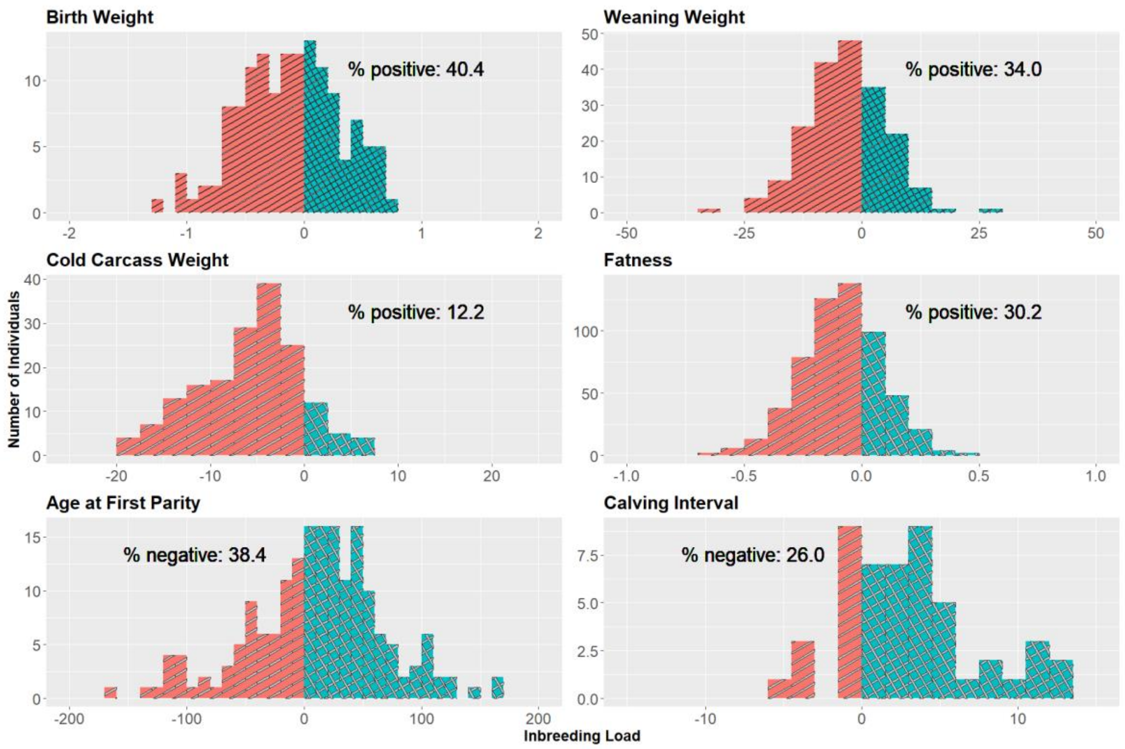

Supplementary Figure 4. Histograms of the predictions of the inbreeding load for Birth Weight, Weaning Weight, Cold Carcass Weight, Fatness, Age at First Parity and Calving Interval for individuals with accuracy over 0.7.

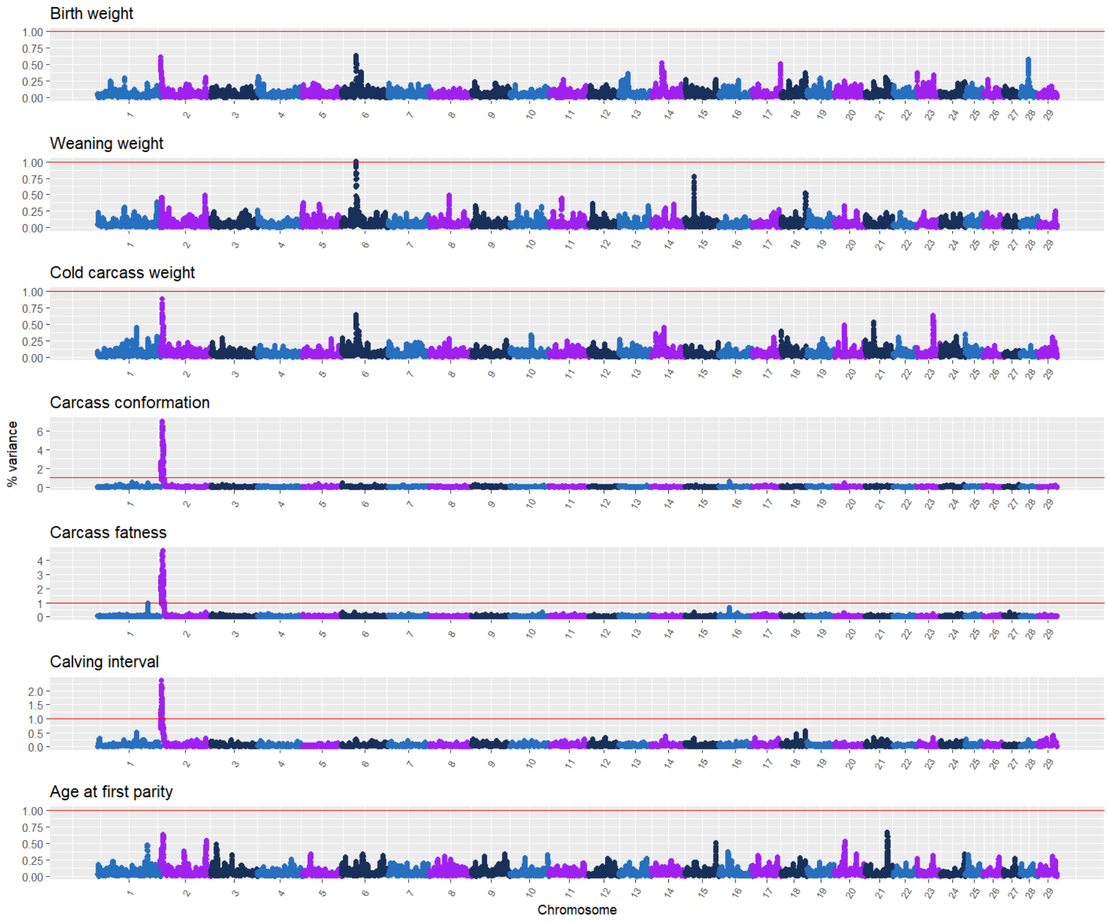

Supplementary Figure 5. Manhattan plots of the percentage of the additive genetic variance explained by segments of 25 SNPs along the autosomal genome for Birth weight, Weaning weight, Cold carcass weight, Carcass conformation, Carcass fatness, Calving interval, and Age at first parity in the Rubia Gallega population.

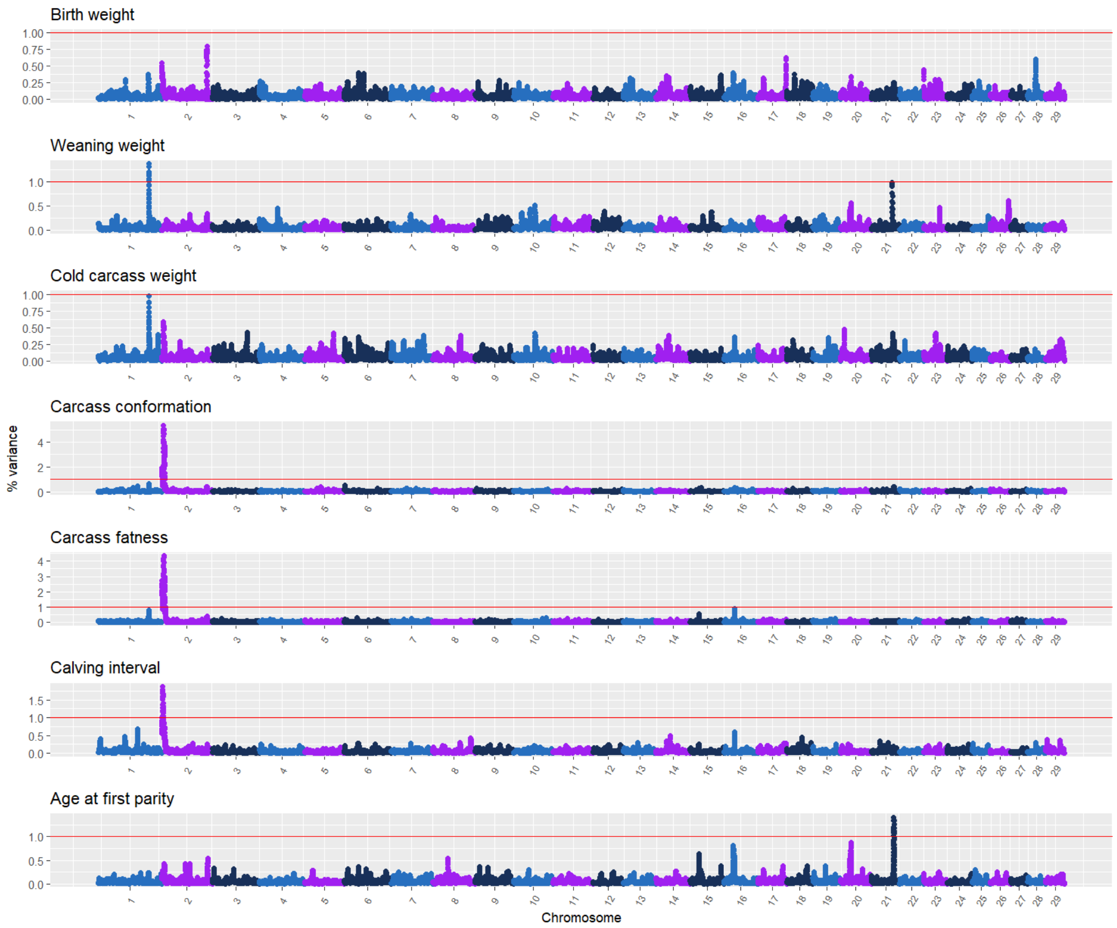

Supplementary Figure 6. Manhattan plots of the percentage of the inbreeding load variance explained by segments of 25 SNPs along the autosomal genome for Birth weight, Weaning weight, Cold carcass weight, Carcass conformation, Carcass fatness, Calving interval, and Age at first parity in the Rubia Gallega population.
