# Supplementary material for: Genomic analysis of the inbreeding load for body weight, carcass and reproductive traits in the Rubia Gallega beef cattle population
Source: Genet Sel Evol. 2026 Mar 13;58:21. doi: 10.1186/s12711-026-01039-8 (PMC13003673; doi:10.1186/s12711-026-01039-8)
Supplement: Supplementary file 2 — Additional File 2 [file 12711_2026_1039_MOESM2_ESM.pdf]

Genomic analysis of the inbreeding load for body weight, carcass and reproductive traits in the Rubia Gallega Beef Cattle population

Carlos Hervás-Rivero, David López-Carbonell, Manuel Sánchez-Díaz, Luis Varona*

Supplementary Table 1. Number of phenotype records for Birth Weigth (BW), Weaning Weight (WW), Cold Carcass Weigth (CCW), Conformation (CONF), Fatness (FAT), Age at First Parity (AFP) and Calving Interval (CI) for the progeny of genotyped sires and dams.

	BW	WW	CCW	CONF	FAT	AFP	CI
Sires	199,738	91,452	80,598	80,551	80,464	40,044	154,887
Dams	9,653	7,836	7,899	7,794	7,887	2,330	7,381

Supplementary Table 2. Number of levels for the systematic and random environmental effects for birth weight (BW), weaning weight (WW), cold carcass weight (CCW), carcass conformation (CONF), carcass fatness (FAT), age at first parity (AFP) and calving interval (CI).

Trait	Mean	Sex	Age of Dam	Slaughterhouse	Parity Order	HYS
BW	1	2	12	-	-	4817
WW	1	2	12	-	-	4238
CCW	1	2	-	37	-	2071
CONF	1	2	-	37	-	2071
FAT	1	2	-	37	-	2071
AFP	1	-	-	-	-	4551
CI	1	-	-	-	15	4722

HYS: Herd-Year-Season.
